# *OsFTL4*, an *FT-like* Gene, Regulates Flowering Time and Drought Tolerance in Rice (*Oryza sativa* L.)

**DOI:** 10.1186/s12284-022-00593-1

**Published:** 2022-09-06

**Authors:** Houwen Gu, Kunming Zhang, Jie Chen, Sadia Gull, Chuyan Chen, Yafei Hou, Xiangbo Li, Jun Miao, Yong Zhou, Guohua Liang

**Affiliations:** 1 Jiangsu Key Laboratory of Crop Genomics and Molecular Breeding/Jiangsu Key Laboratory of Crop Genetics and Physiology/Key Laboratory of Plant Functional Genomics of the Ministry of Education, Yangzhou University, Yangzhou, 225009 China; 2 Jiangsu Co-Innovation Center for Modern Production Technology of Grain Crops, Yangzhou University, Yangzhou, 225009 Jiangsu China; 3 Joint International Research Laboratory of Agriculture and Agri-Product Safety, Yangzhou University, Yangzhou, 225009 China

**Keywords:** Rice, *OsFTL4*, Flowering time, Drought tolerance

## Abstract

The initiation of flowering in cereals is a critical process influenced by environmental and endogenous signals. *Flowering Locus T-like* (*FT-like*) genes encode the main signals for flowering. Of the 13 *FT-like* genes in the rice genome, *Hd3a*/*OsFTL2* and *RFT1*/*OsFTL3* have been extensively studied and revealed to be critical for flowering. In this study, a rice *FT-like* gene, *OsFTL4*, was functionally characterized. Specifically, *osftl4* mutants were generated using a CRISPR/Cas9 system. Compared with the wild-type control (Guangluai 4), the *osftl4-1* and *osftl4-2* mutants flowered 9.6 and 5.8 days earlier under natural long-day and short-day conditions, respectively. Additionally, *OsFTL4* was mainly expressed in the vascular tissue, with the resulting OsFTL4 protein localized in both the nucleus and cytoplasm. Furthermore, OsFTL4 was observed to compete with Hd3a for the interaction with multiple 14-3-3 proteins. An analysis of the effects of simulated drought stress suggested that silencing *OsFTL4* enhances drought tolerance by decreasing stomatal conductance and water loss. These results indicate that OsFTL4 helps integrate the flowering process and the drought response in rice.

**Supplementary Information:**

The online version contains supplementary material available at 10.1186/s12284-022-00593-1.

## Background

In plants, flowering involves a complex physiological process that regulates the transition from the vegetative growth stage to the reproductive growth stage (Sun et al. 2014). An appropriate flowering time is critical for maximizing yield and is influenced by local environmental conditions, including light and temperature (Izawa 2007). After Chailakhyan suggested the existence of a florigen, research involving molecular genetics and molecular biology revealed that the protein encoded by *FLOWERING LOCUS T* (*FT*) is the florigen of plants (Taoka et al. 2013).

The FT protein belongs to the phosphatidylethanolamine-binding protein (PEBP) family, which is commonly found in plants and animals. In plants, *PEBP* genes encode the central regulators of growth and development that control the flowering time, plant architecture, and seed germination (Bradley et al. 1996; Karlgren et al. 2011; Vaistij et al. 2018). The *PEBP* family in angiosperms can be divided into the following three major clades: *FLOWERING LOCUS T* (*FT*), *MOTHER OF FT AND TFL1-like* (*MFT-like*), and *TERMINAL FLOWER1-like* (*TFL1-like*) (Chardon and Damerval 2005). The *MFT-like* gene is mainly expressed in seeds, with the encoded protein affecting the abscisic acid (ABA) and gibberellic acid (GA) signaling pathways to break seed dormancy and promote seed germination as well as flowering (Xi et al. 2010; Nakamura et al. 2011).

Many *FT-like* genes have been identified in the genomes of monocotyledonous crops, including rice (13 *FT* paralogs), wheat and barley (12 *FT* paralogs each), and maize (15 *FT* paralogs) (Halliwell et al. 2016; Danilevskaya et al. 2008; Chardon and Damerval 2005); most of these genes are related to flowering regulation. There are two florigen genes *Hd3a*/*OsFTL2* and *RFT1*/*OsFTL3* in rice, the *Hd3a* more likely induces flowering under short-day (SD), and *RFT1* promotes flowering under long-day (LD) conditions (Kojima et al. 2002; Komiya et al. 2008). These genes are involved in two regulatory pathways: the *OsGI*–*Hd1*–*Hd3a*/*RFT1* pathway is conserved, sharing high similarity with the *GI*-*CO*-*FT* pathway in *Arabidopsis*, and the *Ghd7*–*Ehd1*–*Hd3a/RFT1* pathway is a unique flowering pathway in rice (Izawa et al. 2002; Komiya et al. 2009). Both *Hd3a* and *RFT1* are expressed in the leaf blade vascular tissue, after which the proteins are transported to the SAM through the phloem (Tamaki et al. 2007; Komiya et al. 2009; Pasriga et al. 2018). Previous studies revealed that Hd3a/RFT1 interacts with 14-3-3 proteins in the shoot apical meristem (SAM) cytoplasm and enters the nucleus, wherein it combines with OsFD1 to form a heterohexamer, which is also known as the flowering activation complex (FAC) that induces the expression of the flower development-related genes *OsMADS14* and *OsMADS15* (Taoka et al. 2011; Tamaki et al. 2007). TFL1 is known to antagonize FT proteins, and inhibits the formation of floral primordia in the SAM and delays flowering (Sohn et al. 2007). In rice, the four isoforms of *RICE CENTRORADIALIS* (*RCN*) are homologous to the *TFL1*, which have inhibitory effects on florigen activity (Lifschitz et al. 2014). The RCNs repress flowering by competing with Hd3a for the binding to 14-3-3 proteins and combine with OsFD1 to form a FAC-like heterohexamer complex named the florigen repression complex (FRC) (Kaneko-Suzuki et al. 2018). The overexpression of *RCN1*, *RCN2*, and *RCN3* delays flowering and increases the number of panicle branches (Kaneko-Suzuki et al. 2018; Nakagawa et al. 2002). The balance between FAC and FRC modulates floral initiation to optimize inflorescence development (Kaneko-Suzuki et al. 2018).

Drought stress can alter all aspects of plant growth and development, including the timing of flowering. To adapt to drought conditions, plants often produce seeds before the effects of stress become lethal. In *Arabidopsis*, the activation of *FT* and *TFL1* is induced by GI and ABA under water deficit and LD conditions to promote flowering, whereas under SD conditions, water stress and ABA activate repressors (e.g., SVP) that delay flowering by limiting the transcription of florigen genes (Riboni et al. 2013). Similar to *Arabidopsis*, the drought response in rice is influenced by the ABA signaling pathway. Unlike *Arabidopsis*, the regulation of flowering in rice under drought conditions is not dependent on the photoperiod (Zhang et al. 2016). Earlier research demonstrated that *Ehd1*, which is a photoperiod-related gene, plays a key role in the integration of drought stress and photoperiod signals (Galbiati et al., 2016). Moreover, *Ghd7*, which is expressed upstream of *Ehd1*, is also involved in the regulation of the rice flowering time and drought response (Du et al. 2018). In rice, the overexpression of *OsFTL10* induces flowering and increases drought tolerance (Fang et al. 2019). To date, there is no report describing OsFTL4, which is a homolog of Hd3a, in rice.

In the present study, we characterized an *FT-like* gene, *OsFTL4*, in rice. Knocking out *OsFTL4* via the CRISPR/Cas9 system resulted in an early flowering time under both LD and SD conditions. The *OsFTL4* knockout mutants also exhibited enhanced drought tolerance.

## Results

### Phylogenetic Analysis of Rice *PEBP* Genes

**Fig. 1:**
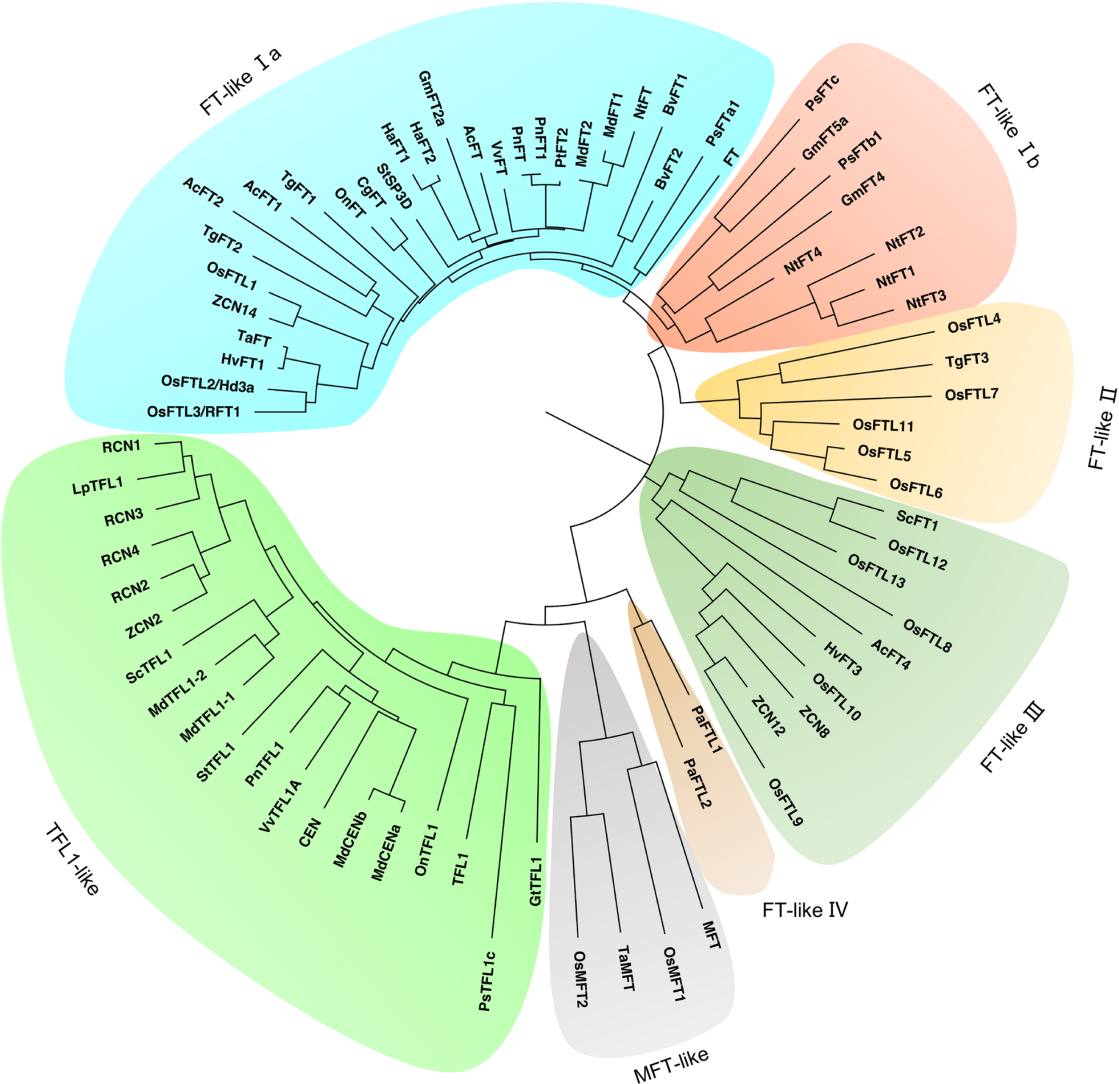
Phylogenetic analysis of *PEBP* family members in *Oryza sativa* and other plant species. Sequence alignment of OsFTL4 and other FT(-like) and TFL1(-like) proteins in *Arabidopsis thaliana*, *Oryza sativa*, *Glycine max*, *Nicotiana tabacum*, *Helianthus annuus*, *Beta vulgaris*, *Tulipa gesneriana*, *Malus domestica*, *Hordeum vulgare*, *Narcissus tazetta*, *Gentia natriflora*, *Oncidium* ‘Gower Ramsey’, *Vitis vinifera*, *Populus nigra*, *Zea mays*, *Picea abies*, *Allium cepa*, *Pisum sativum*, *Ipomoea nil*, *Ananas comosus*, *Populus* spp., *Solanum tuberosum*, *Lolium perenne*, *Cymbidium* spp., *Saccharum* spp., and *Triticum aestivum*

The rice genome contains 19 *PEBP* genes, including 13 *FT-like* genes (*OsFTL1* to *OsFTL13*), 4 *RCN* genes (*RCN1* to *RCN4*), and 2 *MFT* genes (*OsMFT1* and *OsMFT2*). The evolutionary relationship between the 19 *PEBP* genes in rice and the six *PEBP* genes in *Arabidopsis* was investigated (Chardon and Damerval 2005). To clarify the relationship between OsFTL4 and 76 functional PEBP proteins in other species, a neighbor-joining tree was generated after aligning the functionally annotated PEBP proteins from 11 monocotyledonous species (e.g., rice, maize, and onion) and 12 dicotyledonous species (e.g., *Arabidopsis* and soybean) (Fig. 1). The phylogenetic tree included one TFL1-like clade, one MFT-like clade, and five FT-like clades. Most of the TFL1 homologous proteins in the TFL1-like clade repress flowering. The MFT-like clade contained four MFT proteins that vary in terms of their functions. The FT-like clade Ia comprised OsFTL1, OsFTL2/Hd3a, and OsFTL3/RFT1 from rice as well as AtFT from *Arabidopsis*, suggesting that the characteristics of this family in plants developed before the dicot–monocot divergence. Furthermore, most of the *FT-like* genes encode flowering inducers. Although all of the FT-like proteins in FT-like clade Ib are from dicotyledonous species, they have diverse functions. Notably, the FT-like proteins in FT-like clade III, including OsFTL8 and OsFTL10 from rice, ZCN8 and ZCN12 from *Zea mays*, and HvFT3 from *Hordeum vulgare*, are all from monocotyledonous species. Although PaFTL1 and PaFTL2 from *Picea abies* were the only two members of FT-like clade IV in the phylogenetic tree, they delay flowering and appear to be functionally similar to TFL1 (Karlgren et al. 2011). Within clade FT-like II, OsFTL4 and TgFT3, which are highly homologous proteins, were associated with OsFTL5, OsFTL6, OsFTL7, and OsFTL11; however, only TgFT3 has been functionally characterized. The various FT-like proteins from the same species were distributed in different FT-like clusters, indicative of divergence.

### Silencing *OsFTL4* Induces Earlier Flowering in Rice

**Fig. 2:**
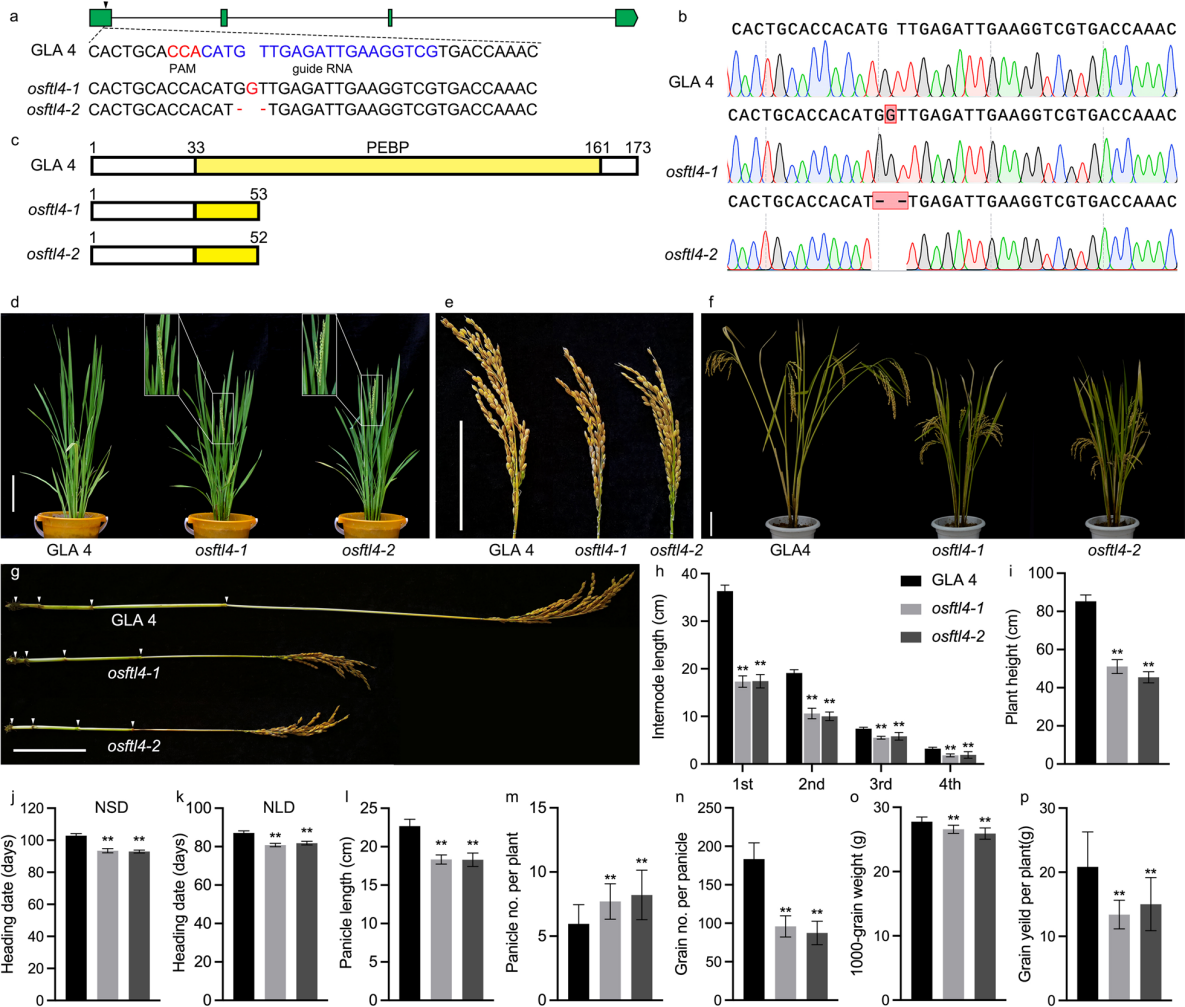
Knockout of *OsFTL4* affects the heading date and other agronomic traits in rice. **a** A specific target guide RNA located in the first exon was used to edit *OsFTL4* via the CRISPR/Cas9 system. The PAM region and guide sequence are marked in red and blue, respectively. The *OsFTL4* mutation sites in the two mutants (*osftl4-1* and *osftl4-2*) are indicated. **b** Mutations were confirmed by sequencing. Mutated bases are marked with red boxes. **c** OsFTL4 protein structures in GLA 4, *osftl4-1*, and *osftl4-2*. The PEBP domain is marked in yellow. **d** Morphology of GLA 4, *osftl4-1*, and *osftl4-2* plants at heading stage. Scale bars, 10 cm. **e** Panicle phenotype of GLA 4, *osftl4-1*, and *osftl4-2*. **f** Morphology of GLA 4, *osftl4-1*, and *osftl4-2* plants at maturity satge. Scale bars, 10 cm. **g** Panicle and internode at maturity. Scale bars, 10 cm. **h** Length of four internodes below the spike. Heading date for GLA 4, *osftl4-1*, and *osftl4-2* in Lingshui **j** and Yangzhou **k**. Comparison between GLA 4 and the mutants in terms of the plant height **i**, panicle length **l**, panicle number per plant **m**, grain number per panicle **n**, 1,000-grain weight **o**, and grain yield per plant **p**. Data are provided as the mean ± SD from 20 individual plants. Significant differences were determined according to Student’s *t*-test (**, *p* < 0.01)

To functionally characterize *OsFTL4*, which is one of the homologs of *Hd3a*, *OsFTL4* knockout mutants with the Guangluai 4 (GLA 4) genetic background (i.e., an early flowering *indica* variety) were generated using the CRISPR/Cas9 system. A total of 10 transgenic lines were obtained. Two homozygous mutants (*osftl4-1* and *osftl4-2*) were isolated from two different transformants and confirmed by sequencing. Compared with the wild-type *OsFTL4* sequence, the *osftl4-1* and *osftl4-2* mutant sequences had a 1-bp insertion and a 2-bp deletion in the target site, respectively (Fig. 2a, b). An amino acid sequence alignment revealed that the changes in the *osftl4-1* and *osftl4-2* sequences were frame-shift mutations that resulted in the introduction of a premature stop codon and the translation of a protein with a truncated PEBP domain (Fig. 2c). The heading date of the two *osftl4* mutants was 9.6 and 5.8 days earlier than that of GLA 4 under natural short-day (NSD) and natural long-day (NLD) conditions, respectively (Fig. 2d, j, k). The *osftl4-1* and *osftl4-2* plants had short panicles (Fig. 2e, l) and a semi-dwarf phenotype at maturity (Fig. 2f, i). Further analyses revealed that the semi-dwarf phenotype of the mutants was due to a decrease in the internode length (Fig. 2g, h). Yield-related parameters were also quantitatively analyzed, including panicle number per plant (PN), grain number per panicle (GN), 1000-grain weight (TGW), and grain yield per plant (GYP) (Fig. 2m–p, Additional file 2: Table S1). The two *osftl4* mutants produced more tillers at the tillering stage (Fig. 2d). At maturity, the PN was 29.4% and 38.0% higher for *osftl4-1* and *osftl4-2*, respectively, than for the wild-type control (Fig. 2m). In contrast, the GN of the mutants decreased significantly (Fig. 2n). Additionally, the TGW of *osftl4-1* and *osftl4-2* decreased by 4.25% and 6.62%, respectively (Fig. 2o). Moreover, there was a significant decrease in the GY of the *osftl4-1* and *osftl4-2* plants (Fig. 2p). Considered together, these findings suggest that *OsFTL4* expression delays flowering and substantially influences multiple agronomic traits in rice.

### *OsFTL4* Expression Pattern and Subcellular Localization of the Encoded Protein

**Fig. 3:**
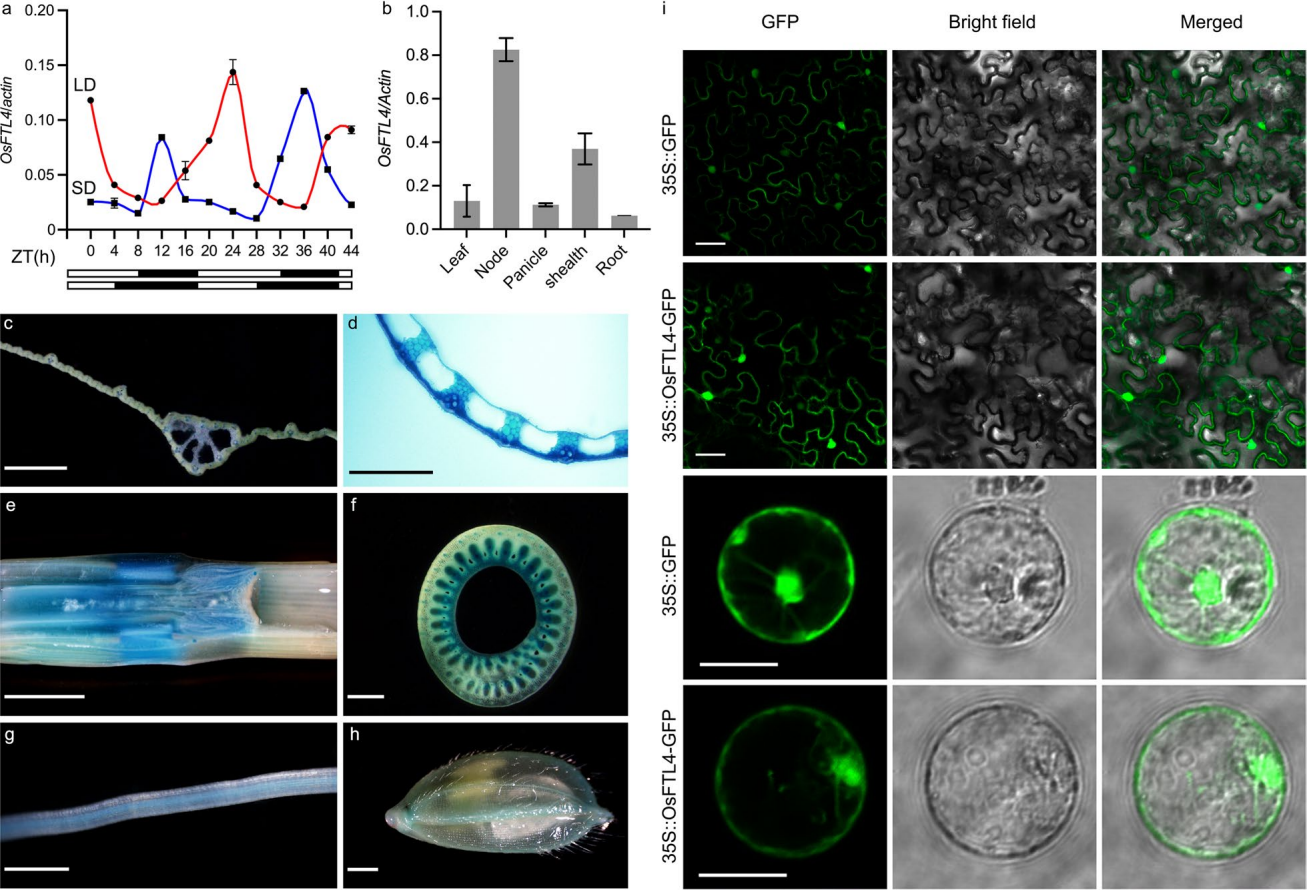
*OsFTL4* expression profile. **a** Diurnal *OsFTL4* expression pattern under SD and LD conditions. **b** *OsFTL4* transcript levels in different tissues. **c** *OsFTL4* promoter-GUS activity was detected in the leaf. **d** Sheath. **e** Node. **f** Stem. **g** Root. **h** Mature spikelet before fertilization. Scale bars: **c** 0.5 mm; **e** 5 mm; **d**, **f**–**h** 1 mm. **i** Subcellular localization of OsFTL4 in *Nicotiana benthamiana* leaf epidermal cells and rice protoplasts. Scale bars, 50 μm in *N. benthamiana* and 20 μm in rice

To examine the temporal and spatial expression patterns of *OsFTL4*, we performed a quantitative real-time PCR (qRT-PCR) analysis to examine *OsFTL4* expression levels in various tissues collected from GLA 4 plants grown under NLD conditions. The qRT-PCR data indicated that *OsFTL4* was expressed in all examined tissues, but especially in the node and sheath (Fig. 3b). The tissue-specific expression was analyzed using *OsFTL4* promoter-GUS transgenic plants. Consistent with our qRT-PCR results, the GUS signal was detected in the panicle, node, root, sheath, and leaf blade (Fig. 3c–h).

We also investigated the *OsFTL4* expression pattern under SD (10-h light, 28 °C/14-h dark, 26 °C) and LD (14-h light, 28 °C/10-h dark, 26 °C) conditions in an artificial climate chamber. Diurnal rhythms in *OsFTL4* transcription were detected, they differed between the controlled short-day (CSD) and controlled long-day (CLD) conditions, and *OsFTL4* is upregulated during the light period under CLD and the dark period under CSD (Fig. 3a). *Arabidopsis* lacks a homolog of *Ehd1*, which encodes a B-type response regulator. *Ehd1*–*Hd3a*/*RFT1* is a unique flowering pathway in rice, in which *Ehd1* induces *Hd3a*/*RFT1* expression under SD and LD conditions to promote flowering (Doi et al. 2004). *Ehd1* exhibited a circadian pattern in *osftl4* plants under both LD and SD conditions. Additionally, there was no difference in the transcription of *OsphyB* and *OsGI* between the wild-type and *osftl4* plants, indicating that *OsFTL4* is expressed downstream of *OsphyB* and *OsGI* (Additional file 1: Fig. S1). Furthermore, *OsFTL4* regulates flowering as part of the *Ehd1* pathway, possibly via the feedback-regulated expression of *Ehd1.*

On the basis of the ProtComp online tool (http://www.softberry.com/), the OsFTL4 protein was predicted to be localized in both the cytoplasm and nucleus. To verify the predicted localization, the OsFTL4-green fluorescent protein (GFP) fusion construct under the control of the CaMV 35S promoter was generated. The recombinant 35S:: OsFTL4-GFP construct was transiently expressed in *Nicotiana benthamiana* leaf epidermal cells and rice protoplasts. Confocal microscopy images confirmed that OsFTL4 was localized in the cytoplasm and nucleus (Fig. 3i).

### Regulatory Effects of *OsFTL4* on the Heading Date

**Fig. 4:**
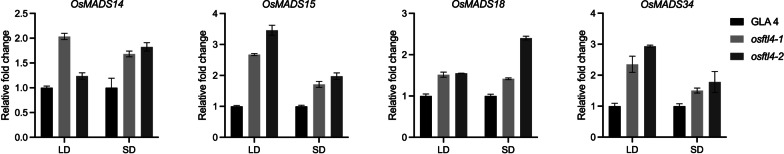
Comparison of floral meristem identity gene transcription levels of the wild-type and mutant plants. *OsMADS14*, *OsMADS15*, *OsMADS18*, and *OsMADS34* expression levels in GLA 4 and *osftl4* under LD and SD conditions. For all examined genes, the transcription level in GLA 4 was set at 1 (i.e., baseline). Each point represents three technical replicates

To reveal the possible *OsFTL4* genetic network, we compared the expression levels of several rice genes that control flowering. Among these genes, the *Sepallata* (*SEP*) gene *OsPAP2*/*OsMADS34* and the three *AP1*/*FUL-like* genes *OsMADS14*, *OsMADS15*, and *OsMADS18* encode florigen signals in the meristem (Preston and Kellogg 2006; Kobayashi et al. 2012). The *OsMADS14*, *OsMADS15*, *OsMADS18*, and *OsMADS34* transcription levels were higher in the two *osftl4* mutants than in GLA 4 under both LD and SD conditions (Fig. 4). Accordingly, *OsFTL4* may delay flowering by downregulating the expression of *OsMADS14*, *OsMADS15*, *OsMADS18*, and *OsMADS34*.

### OsFTL4 Competes with Hd3a for the Interaction with 14-3-3 Proteins

**Fig. 5:**
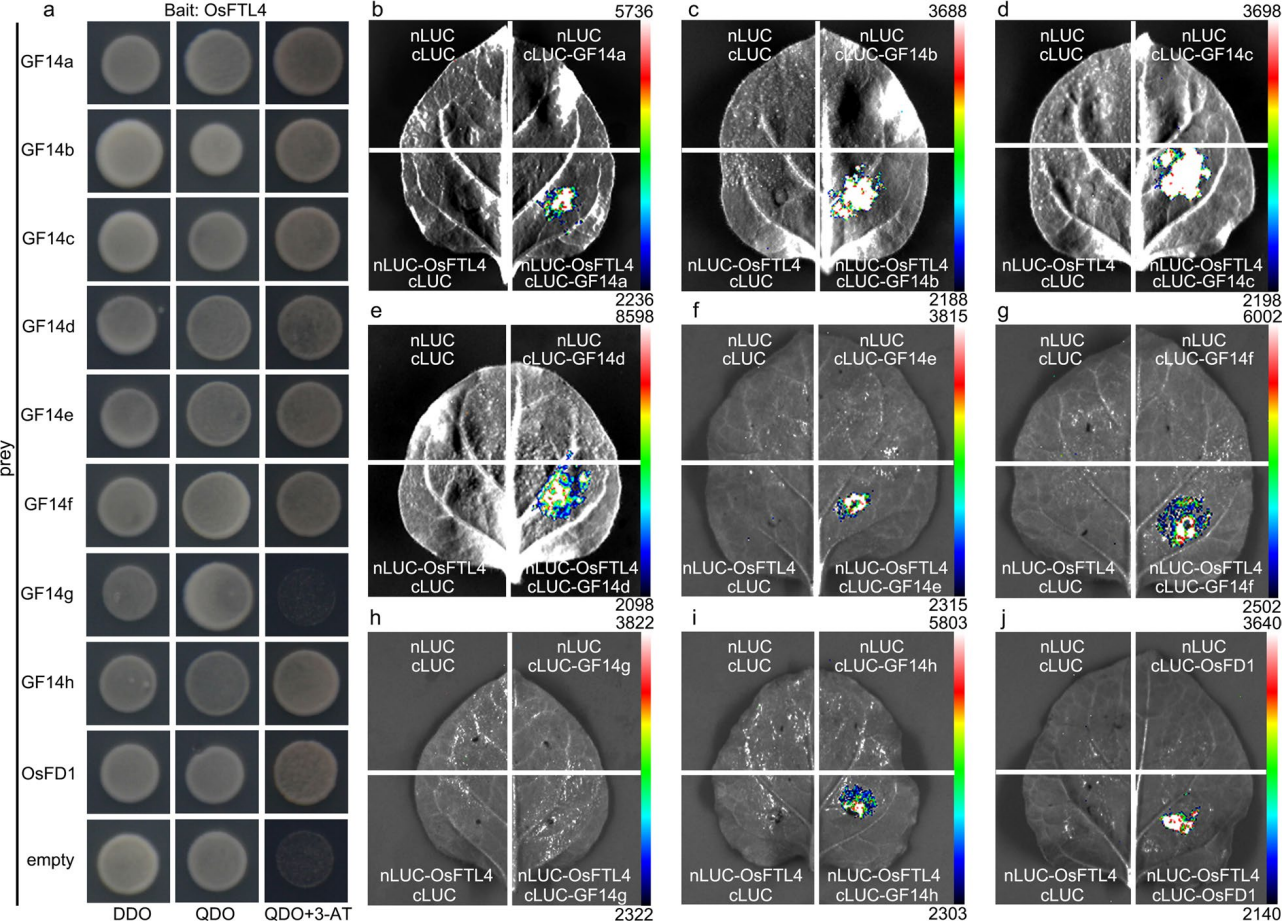
Interactions among OsFTL4, 14-3-3, and OsFD1 proteins. **a** Y2H assay. OsFTL4 was used as bait (GAL4-BD, BD), whereas GF14a to GF14h and OsFD1 were used as prey (GAL4-AD, AD). DDO: SD/-L/-T, QDO: SD/-L/-T/-H/-A. The 3-AT concentration was 25 mM. The interaction between OsFTL4 and GF14a **b**, GF14b **c**, GF14c **d**, GF14d **e**, GF14e **f**, GF14f **g**, GF14g **h**, GF14h **i**, or OsFD1 **j** was determined by the LCI assay involving *N. benthamiana* leaf epidermal cells. Effect of OsFTL4 on the activation of *OsMADS15* expression by FAC. Data are presented as the mean ± SEM of three independent experiments

The interaction between Hd3a and 14-3-3 proteins in the SAM generates a complex that is translocated to the nucleus, where it binds to OsFD1. The resulting ternary FAC complex induces the transcription of *OsMADS15*, which leads to flowering (Taoka et al. 2011). To elucidate the mechanism underlying the inhibitory effects of OsFTL4 on flowering, the interaction of OsFTL4 with 14-3-3 proteins and OsFD1 was analyzed by conducting a yeast two-hybrid (Y2H) assay. Specifically, *OsFTL4* was inserted into pGBKT7, whereas sequences encoding eight 14-3-3 isoforms (GF14a to GF14h) and OsFD1 were inserted into pGADT7. All combinations of recombinant plasmids were used to transform Y2HGold yeast cells. The Y2H assay confirmed that OsFTL4 can interact with GF14a, GF14b, GF14c, GF14d, GF14e, GF14f, GF14h, and OsFD1 in yeast cells (Fig. 5a). However, there was no interaction between OsFTL4 and GF14g. We also examined the interactions in a luciferase complementation imaging (LCI) assay involving *N. benthamiana* leaves, which produced similar results (Fig. 5b–j).

**Fig. 6:**
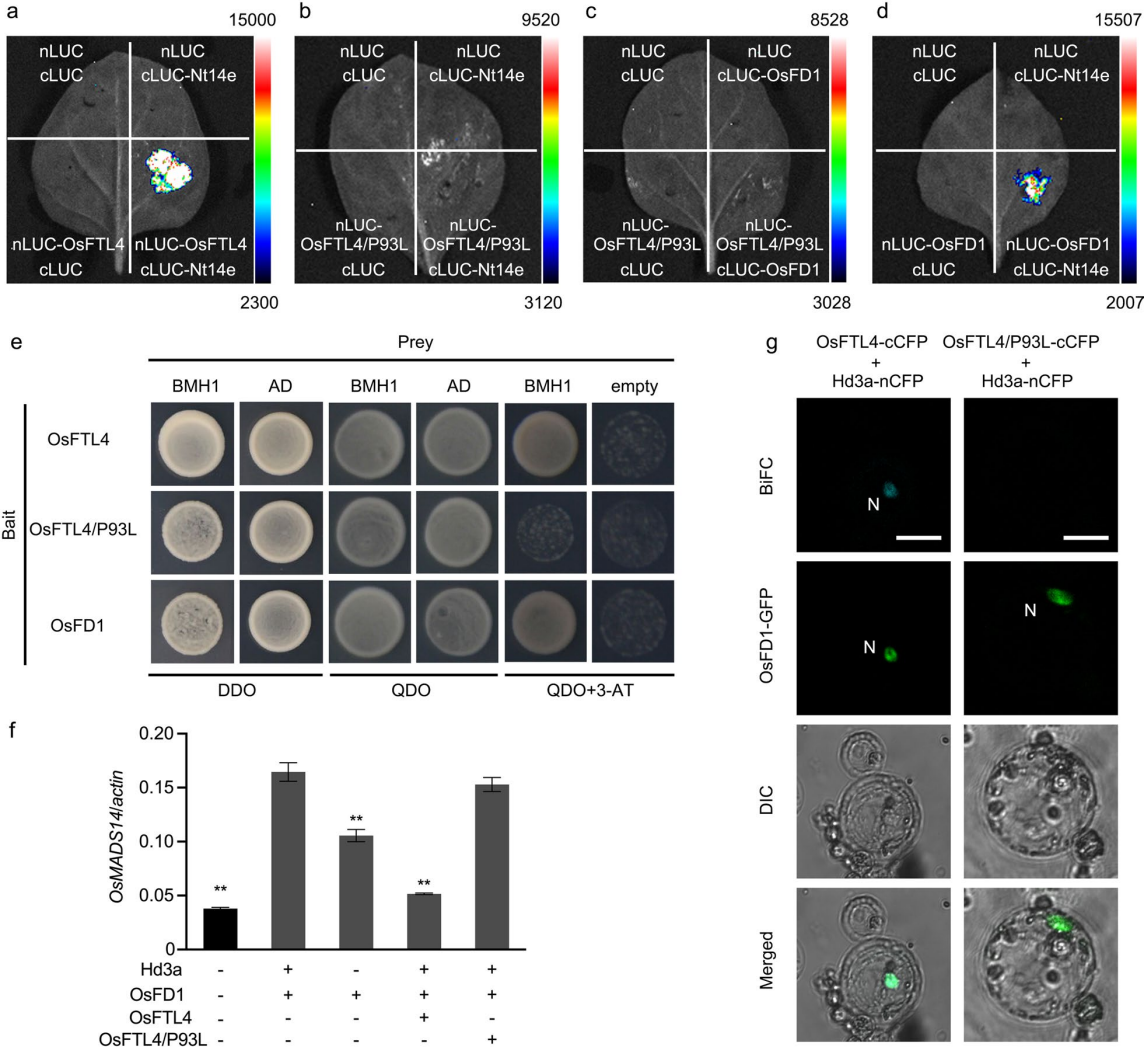
OsFTL4 competes Hd3a for formation of florigen activation complex. The interaction between OsFTL4 and Nt14-3-3e **a**, OsFTL4/P93L and Nt14-3-3e **b**, OsFTL4/P93L and OsFD1 **c**, OsFD1 and Nt14-3-3e **d** were detected by using LCI assays. Nt14e: Nt14-3-3e. **e** The interaction between Nt14-3-3e and OsFTL4, Nt14-3-3e and OsFTL4/P93L, OsFD1 and Nt14-3-3e were tested by using Y2H assays. **f** Effect of OsFTL4 on *OsMADS14* activation by FAC. Values are means ± SEM from three independent experiment. Asterisks indicate the significance of differences determined by Student’s *t* test: **, *p* < 0.01. **g** Co-localization of Hd3a and OsFTL4. BiFC assays between Hd3a and OsFTL4 (left) and between Hd3a and OsFTL4/P93L (right) were performed. N: Nucleus. Scale bar: 20 μm

OsFD1 and Hd3a do not interact in vitro, whereas the presence of interactions in the yeast system is due to the bridging role played by the yeast 14-3-3 protein BMH1(Taoka et al. 2011). In the present study, OsFTL4 could interact with OsFD1 in yeast and tobacco system. Several 14-3-3 isoforms were present in tobacco, and some of them were reported to interact with bZIP proteins (Igarashi et al. 2001). Meanwhile, these interactions were dependent on conserved residuals (Taoka et al. 2011; Igarashi et al. 2001). Protein sequence alignment revealed that Nt14-3-3a, Nt14-3-3b, Nt14-3-3e in tobacco and brain modulosignalin homolog 1 (BMH1) in yeast were highly similar to the rice 14-3-3 proteins OsGF14b and OsGF14c sharing the same conserved residuals (Additional file 1: Fig. S2). Therefore, the interaction between BMH1 with OsFTL4 and OsFD1, Nt14-3-3e with OsFTL4 and OsFD1were verified by Y2H assay and LCI assay, respectively (Fig. 6a–e). Furthermore, OsFTL4/P93L, a key site mutantion of OsFTL4, could not interact with BMH1, Nt14-3-3e and OsFD1 (Fig. 6b, c, e).

To test the hypothesis that OsFTL4 and RCN are functionally similar, i.e., RCN competes with Hd3a for the binding to 14-3-3 proteins to form FAC (Kaneko-Suzuki et al. 2018), we co-expressed *Hd3a* and *OsFD1* in rice protoplasts. This co-expression activated the expression of *OsMADS14*. Meanwhile, single transformation of OsFD1 also activated the expression of *OsMADS14*. However, its activation was significantly diminished when *OsFTL4* was co-expressed, and no reduction in *OsMADS14* expression levels was observed when *OsFTL4*/*P93L* mutant was co-expressed (Fig. 6f). These results demonstrated that OsFTL4 competes with Hd3a for the interaction with 14-3-3 proteins to repress the floral transition in rice. To further explore the competitive relationship between OsFTL4 and Hd3a, we performed BiFC analysis between OsFTL4 and Hd3a. When Hd3a-nCFP, OsFTL4-cCFP fusion proteins were co-expressed with OsFD1 in rice protoplasts, cyan fluorescent signals of Hd3a-OsFTL4 interactions were observed in the nucleus. When a P93L substitution was introduced in OsFTL4, no BiFC signal was detected (Fig. 6g).

### Mutation to *OsFTL4* Improves Rice Drought Tolerance

**Fig. 7:**
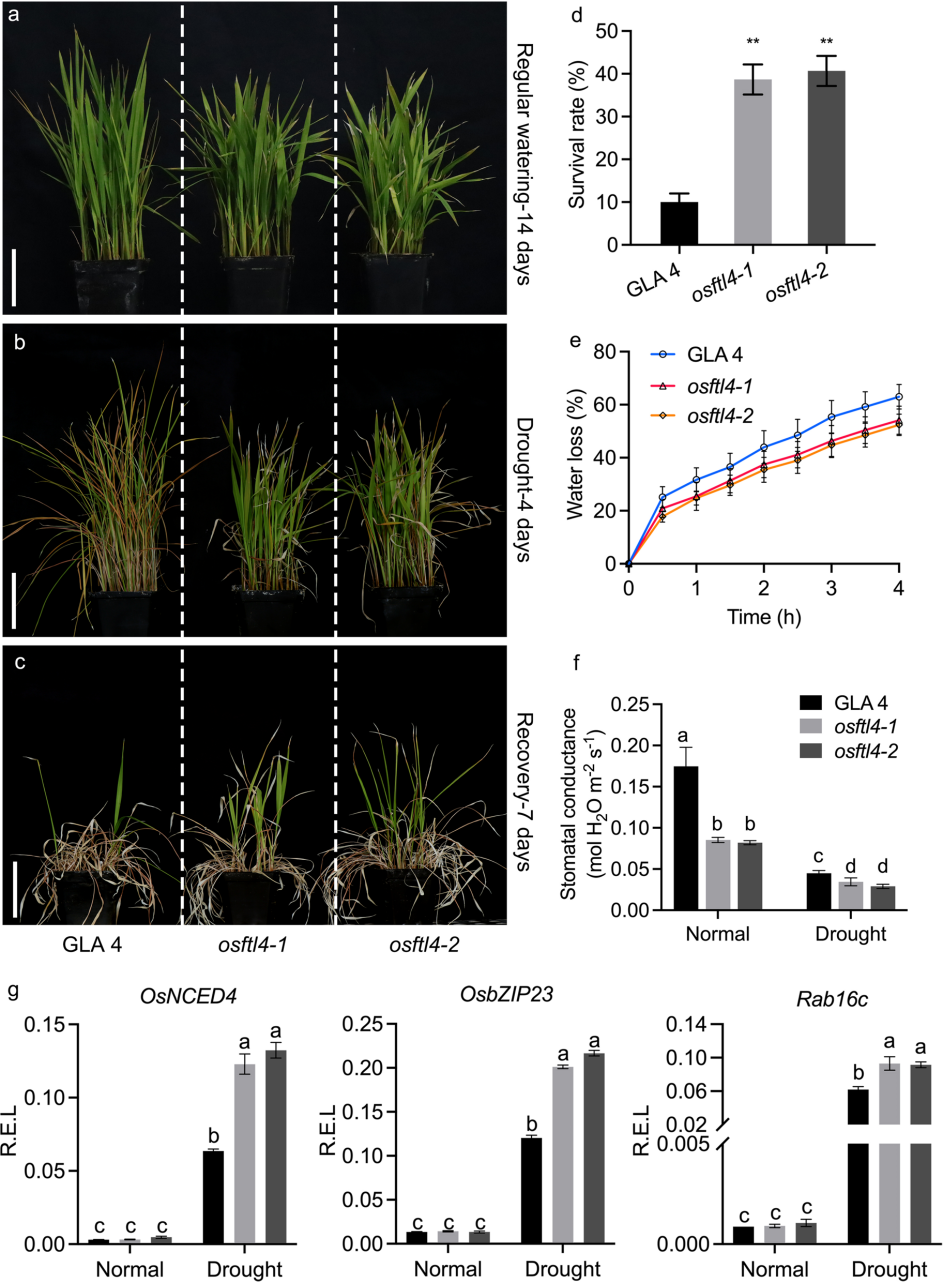
Phenotypes of GLA 4 as well as *osftl4-1* and *osftl4-2* mutants under drought conditions. **a**–**c** Both mutants (*osftl4-1* and *osftl4-2*) exhibited enhanced drought tolerance. The GLA 4, *osftl4-1*, and *osftl4-2* seedlings (nearly 40 seedlings per genotype) with regular watering for 14 days **a** were subjected to drought stress for 4 days **b**, and recovery for 7 days **c**, respectively. Scale bars, 5 cm. **d** Survival rate calculated on the basis of the growth of a new leaf blade after 7 days of re-watering. Error bars represent the SE of three biological replicates (**, *p* < 0.01, by Student’s *t*-test). **e** Water loss rate of detached leaves from the wild-type and mutant lines at different time-points. Data are presented as the mean ± standard deviation (n = 20). **f** Stomatal conductance of the wild-type and two mutant lines under normal and drought stress conditions. Error bars represent three biological replicates (six plants per replicate). **g** *OsNCED4*, *OsbZIP23*, and *Rab16c* expression levels in wild-type and *osftl4* plants under normal and drought conditions. Mean values ± SD were obtained from three technical repeats and three biological repeats. Statistical differences are labeled with different letters using LSD test (*p* < 0.05, one-way ANOVA)

An examination using the PLACE online program (http://www.dna.affrc.go.jp/PLACE) suggested that the *OsFTL4* promoter region contains multiple hormone-responsive elements (Additional file 3: Table S2). A previous study determined that ABA helps increase the expression of drought-responsive genes in rice (Rabbani et al. 2003). We observed that *OsFTL4* expression was repressed by ABA (Additional file 1: Fig S3). To investigate whether *OsFTL4* is involved in rice responses to drought stress, the drought tolerance of the *osftl4* mutants was assessed. The wild-type plants and *osftl4* mutants were grown under well-watered conditions for 2 weeks (Fig. 7a), after which watering was stopped. Following a 4-day exposure to drought stress, all of the wild-type GLA 4 plants were severely affected, whereas the *osftl4* mutant plants exhibited less leaf rolling and wilting (Fig. 7b). After the recovery period, *osftl4* mutant plants had more green leaves than the wild-type plants (Fig. 7c). Additionally, the survival rates of the two *osftl4* mutants (38.7% and 40.7%) were significantly higher than that of the wild-type plants (Fig. 7d).

The results of an in vitro water dissipation test involving the leaves of 2-week-old GLA 4 and *osftl4* plants indicated that the rate of water dissipation was significantly lower in the mutants than in the wild-type control plants (Fig. 7e). The stomatal status is an important factor associated with plant drought responses. Thus, the stomatal conductance of the wild-type and *osftl4* plants at the 4- to 5-leaf stage under normal and drought conditions was analyzed. Drought stress obviously affected stomatal closure, leading to decreased conductance, but the stomatal conductance of the *osftl4* mutant plants was significantly lower than that of the wild-type plants (Fig. 7f).

To explore the possible molecular mechanisms by which *OsFTL4* negatively regulates drought tolerance in rice, the expression of several stress-responsive genes under normal and drought conditions was analyzed. These genes include *OsNCED4*, encoding a protein involved in ABA biosynthesis (Zhu et al. 2009); *OsbZIP23*, encoding a basic leucine zipper protein (Zong et al. 2016); *Rab16c*, encoding a late embryogenesis abundant protein (LEA) (Xiao et al. 2007). Compared with their expression under normal conditions, *OsNCED4*, *OsbZIP23*, and *Rab16c* expression levels increased in the wild-type and mutant plants in response to drought stress. However, these genes were more highly expressed in the *osftl4* mutants than in the GLA 4 plants under drought conditions (Fig. 7g). These findings imply that a mutation to *OsFTL4* enhances drought tolerance by inducing drought stress-responsive gene expression in rice.

## Discussion

The FT-like proteins belong to the PEBP family, which is widely conserved among plant species (Bradley et al. 1996). The function of *Hd3a*/*RFT1*, the main genes in the *FT-like* family, during the floral transition of rice has been well studied. However, the functions of the other members of this family in rice remain relatively uncharacterized. In this study, knocking out *OsFTL4* resulted in earlier flowering under SD and LD conditions and enhanced the drought tolerance of rice. In contrast, the *osftl4* knockout mutation resulted in a shorter rice plant, a shorter panicle, and fewer grains per panicle, which ultimately led to a decreased grain yield.

In rice, an appropriate flowering time is important for enhancing regional adaptations and increasing grain yield. One of the most essential environmental cues for flowering is the photoperiod. Photoperiodic flowering is regulated by a combination of light signals and circadian rhythms (Song et al. 2015). With dual functions, *Hd1* promotes flowering under SD conditions, but suppresses flowering under LD conditions (Izawa et al. 2002). Unlike *Hd1*, *Ehd1* promotes flowering under LD conditions (Wei et al. 2016). A previous study revealed that Ghd7 forms a complex with Hd1 to repress *Hd3a* transcription, while also substantially repressing *Ehd1* expression to delay flowering under LD conditions in rice (Nemoto et al. 2016). Another study indicated that a mutation to *OsELF3*/*Ef7*, which is a homolog of *Arabidopsis ELF3*, significantly increases the transcription of *Ghd7* to delay flowering under both LD and SD conditions (Yang et al. 2013). *RFT1* is predominantly expressed under LD conditions and *Hd3a* is the major floral activator under SD conditions in rice. In response to the regulatory effects of the photoperiod, both *Hd3a* and *RFT1* are transcribed; the silencing of both genes via RNAi leads to not flower even at 300 days after sowing (Komiya et al. 2008). To date, no *FT-like* gene has been reported to have the same function in both SD and LD conditions. In this study, *OsFTL4* knockout mutants flowered earlier than the wild-type control under both NLD and NSD conditions (Fig. 2). GLA 4 is a semi-dwarf early rice variety that is photoperiod insensitive and temperature sensitive. Therefore, based on the genetic background, *OsFTL4* may be a genetic resource for heading time and plant height improvement in some tall-stalked late rice varieties. Additionally, *OsFTL4* expression is induced by darkness under CSD conditions (Fig. 3a), which may explain the longer delay in the flowering of the *osftl4* mutant plants under NSD conditions than under NLD conditions (Fig. 2j, k).

The qRT-PCR and GUS staining results indicated that *OsFTL4* was constitutively expressed and primarily distributed in vascular bundle tissues (Fig. 3b, c–k), which was different from the expression pattern of *Hd3a* and *RFT1* (Komiya et al. 2008). During flower formation, *Hd3a*/*RFT1* is expressed in leaves, with the resulting protein transported to the shoot apex through vascular bundles to induce flowering (Tamaki et al. 2015, 2007; Komiya et al. 2008). The *RCN* genes, which encode flowering suppressors, are mainly expressed in the stem during the vegetative, transition, early reproductive, and late reproductive phases (Kaneko-Suzuki et al. 2018). The OsFTL4 protein is located in both the cytoplasm and nucleus (Fig. 3l), which is similar to all charaterized *FT-like* genes (Zhang et al. 2020; Fang et al. 2019; Zhan et al. 2017). A recent study detected the RCN3 and 14–3-3 BiFC signals in the cytoplasm and nucleus (Kaneko-Suzuki et al. 2018). In rice, RCN proteins, which compete with Hd3a, interact with 14-3-3 proteins (GF14b, GF14c, GF14e, and GF14f) in the cytoplasm and are then transferred into the nucleus to interact with OsFD1, a rice FD homologous protein, resulting in the formation of the FRC complex that decreases florigen activity (Kaneko-Suzuki et al. 2018; Taoka et al. 2011). Moreover, in rice, OsFTL10 functions as a floral inducer that can interact with OsFD1 as well as various 14-3-3 homologs, including GF14a, GF14b, GF14c, GF14d, and GF14e (Fang et al. 2019). In the current study, OsFTL4 was observed to interact with OsFD1 and GF14a, GF14b, GF14c, GF14d, GF14e, GF14f, and GF14h in yeast and *Nicotiana benthamiana* cells (Fig. 5a, b-j). However, the interaction between OsFTL4 and OsFD1 required the presence of 14-3-3 proteins in yeast and tobacco cells (Fig. 6a–e). Thus, FT-like may regulate flowering by interacting with different 14-3-3 isoforms in rice. In addition, competition assays showed that OsFTL4 inhibits flowering by competing with Hd3a to form an FRC-like complex (Fig. 6f, g). OsFTL4 functions similarly to RCN (Kaneko-Suzuki et al. 2018).

Drought is an environmental stress that influences crop growth. Unlike animals, plants are sessile organisms. Plants mitigate the adverse effects of drought stress by regulating their vegetative and reproductive growth according to water availability (Shim and Jang 2020). Mild drought conditions can accelerate flowering in rice (Du et al. 2018). Stomatal closure can decrease plant water loss and help plants maintain the tissue water potential following the uptake of water from the soil through deep-growing roots (i.e., drought avoidance) (Hu and Xiong 2014). In this study, compared with the wild-type plants, the water loss and stomatal conductance decreased in the *osftl4* mutant plants following an exposure to drought stress (Fig. 7a–d). Moreover, *Hd3a*, *RFT1*, and *Ehd1* appear to integrate the photoperiod and drought stress signals to delay rice floral transition. Drought stress reportedly significantly decreases the transcription of *Ehd1*, *Hd3a*, and *RFT1* (Galbiati et al. 2016). Drought-induced flowering occurs via the ABA-dependent upregulated expression of the two florigen genes *Hd3a* and *RFT1*. Similarly, ABA can repress *OsFTL4* transcription (Additional file 1: Fig. S3). The ABA-induced upregulation of *Hd3a* and *RFT1* expression involves the ABA-induced expression of *OsbZIP23*. The overexpression of *OsbZIP23* was revealed to upregulate and downregulate *Ehd1* and *Ghd7* expression levels, respectively (Du et al. 2018). Drought also induces the expression of the circadian clock genes *OsGI* and *OsTOC1*, which encode positive regulators of *Hd3a* and *RFT1* expression that promote flowering (Riboni et al. 2013; Valim et al. 2019). Furthermore, RCN1 has antagonistic effects on the florigen. A recent investigation determined that *rcn1* mutant plants flower early and are relatively insensitive to drought stress (Wang et al. 2020). Additionally, it has been demonstrated that stomatal conductance and photosynthetic rate are positively correlated and ultimately influence plant growth and yield through affecting CO_2_ assimilation (Kusumi et al. 2012). Some high-yielding cultivars, such Takanari (Xu et al. 1997) and Habataki (Adachi et al. 2011), have a high stomatal conductance and photosynthetic rate. In the current study, the reduced stomatal conductance of the *osftl4* mutant compared to the wild type may have reduced CO_2_ uptake by reducing leaf photosynthetic rate, resulting in reduced plant growth and lower grain yield.

## Conclusion

In this study, we identified a novel *FT-like* gene, *OsFTL4*. In contrast to FT, *OsFTL4* represses flowering in rice and integrates the mechanisms mediating flowering and drought tolerance.

## Materials and Methods

### Plant Materials and Growth Conditions

GLA 4, which is an *indica* cultivar, was used to generate transgenic lines. The wild-type and knockout mutants were grown in the transgenic experimental field of Yangzhou University, China. Pure lines were screened and grown in transgenic experimental fields under NLD conditions at Yangzhou University (119.43° E, 32.39° N) and NSD conditions in Lingshui, Hainan, China (110.05° E, 18.51° N). During the floral transition period of rice, the day length was longer than 13.5-h in Yangzhou and shorter than 12.5-h in Lingshui (Additional file 1: Fig. S4) (The Data was collected from www.timeanddate.com).

### Phylogenetic Analysis

The PEBP family proteins were aligned using Clustal Omega (https://www.ebi.ac.uk/Tools/msa/clustalo). The aligned sequences were used for the phylogenetic analysis, which was performed according to the neighbor-joining method of MEGA X (Kumar et al. 2018). The phylogenetic tree was modified and annotated using FigTree (version 1.4.4).

### Vector Construction and Rice Transformation

To construct the CRISPR-Cas9 vector for *OsFTL4*, the predictions made by CRISPR-GE (http://skl.scau.edu.cn/) were used to select a guide sequence targeting the first exon with a low off-target rate. The gRNA framework with the guide sequence was inserted into the pC1300-Ubi:Cas9 empty vector to produce the CRISPR-Cas9 recombinant vector (Hu et al. 2018). The 2.0-kb *OsFTL4* promoter sequence (i.e., sequence upstream of the ATG start codon) was amplified by PCR using GLA 4 genomic DNA as the template and then inserted into the pCAMBIA1301 vector to control the expression of the GUS-encoding gene. All constructs were introduced into *Agrobacterium tumefaciens* EHA105 cells via chemical transformation and then inserted into GLA 4 plants using a previously described *A. tumefaciens*-based method (Hiei et al. 1994). All primers used for constructing recombinant vectors are listed in Additional file 4: Table S3.

### Histochemical Analysis of GUS Activity

For the GUS staining analysis, leaves, nodes, sheaths, stems, roots, and young panicles were collected from hygromycin-resistant transformed plants at the booting stage. The GUS activity was detected using the GUSblue kit (Huayueyang, Beijing, China) according to the manufacturer's manual. The samples were destained with pure ethanol and then examined.

### Subcellular Localization

The *OsFTL4* coding sequence was amplified by PCR and incorporated into pAN580 and pHG to form pAN580-OsFTL4 and pHG-OsFTL4, respectively. For the transient expression assay in rice protoplasts, the pAN580 empty vector and the pAN580-OsFTL4 recombinant vector were inserted into separate rice protoplasts as previously described (Chen et al. 2006). *Agrobacterium tumefaciens* GV3101 cells were transformed with the pHG empty vector or the pHG-OsFTL4 recombinant vector for the subsequent infiltration of healthy *N. benthamiana* leaves. Fluorescent signals were detected using the Zeiss LSM 710 laser scanning confocal microscope. The primers used for constructing recombinant vectors are listed in Additional file 4: Table S3.

### Transient Expression Assay in Rice Protoplasts

For the transient expression analysis, different combinations of 4 μg pAN580-Hd3a, 4 μg pAN580-FTL4, 4 μg pAN580-FTL4/P93L and 5 μg pAN580-OsFD1 recombinant vectors were inserted into 100 µl rice protoplasts according to a polyethylene glycol (PEG)-mediated method (Zhang et al. 2011). After a 16-h incubation at 25 °C, the protoplast suspension was centrifuged and the cell pellet was collected for an RNA extraction. The cDNA synthesized via reverse transcription was used for the qRT-PCR analysis.

### Diurnal Expression Analysis

Rice plants grown under natural day-length conditions for 4 weeks were transferred to a growth chamber and incubated under SD (10-h light, 28 °C/14-h dark, 26 °C) or LD (14-h light, 28 °C/10-h dark, 26 °C) conditions with 65% relative humidity. Seven days later, the leaves of each line were harvested every 4 h for 48 h. For each time-point, the leaves from three different individuals were collected as biological replicates.

### ABA Treatment Assays

To detect the response of *OsFTL4* to ABA, 2-week-old seedlings of GLA4 were placed into -ABA or ABA solution in the light. For ABA dose-dependent tests, ABA concentration were set at 0 μΜ, 1 μΜ, 5 μΜ, 10 μΜ, 30 μΜ, and 50 μΜ, and expression levels of *OsFTL4* were detected after 3-h of ABA treatment. Furthermore, transcript levels of *OsFTL4* were also detected at 0-h, 3-h, 6-h, 9-h, and 12-h after treatment under 10 μΜ ABA conditions.

### Drought Tolerance Assays

GLA 4 and the two mutant lines were grown in a growth chamber (12-h light, 28 °C/12-h dark, 26 °C) for 2 weeks. The second leaves of the wild-type and two *osftl4* mutants were selected for the water loss rate test. The samples were weighed every 30 min and the water loss rate was calculated using the following formula: water loss rate (%) = (fresh weight − dry weight)/fresh weight × 100. For drought treatment, 2-week-old rice seedling were exposed to drought stress treatments. Each pot was filled with the same amount of fluffy and breathable nutrient soil. The water was withheld for 7 days and then the drought stressed plants were re-watered to recover. After recovery, the survival rates (%) were calculated from the numbers of surviving plants with new leaves appeared.

### Stomatal Conductance Analysis

The second leaves of 3-week-old rice plants were selected for the stomatal conductance analysis, which was performed using the LI-6400XT Portable Photosynthesis System (LI-COR, USA) before and after a 3-day exposure to drought conditions.

### RNA Extraction and Quantitative Real-Time PCR

Total RNA was extracted from diverse tissues collected from plants exposed to different treatment conditions using an RNA simple Total RNA Kit (Tiangen, Beijing, China) according to the manufacturer's manual and then total RNA was reverse transcribed into cDNA using the Fast King One-step RT-qPCR kit (Tiangen, Beijing, China). The qRT-PCR analysis was performed using the ABI Viia™ system (Life Technologies, USA) and the AceQ qPCR SYBR Green Master Mix (Vazyme, Nanjing, China). Details regarding the qRT-PCR primers are listed in Additional file 4: Table S3.

### Yeast Two-Hybrid Assay

The coding sequences of eight 14-3-3 isoforms (*OsGF14a* to *OsGF14h*) were PCR-amplified from rice cDNA and cloned into separate pGADT7 vector, the full-length *OsFTL4* sequence was inserted into the pGBAD7 and pGBKT7 vector, respectively. The OsFTL4/P93L-BD mutant vector was obtained by single-base mutation of OsFTL4-BD vector. The full-length *BMH1* sequence was PCR-amplified from yeast and inserted into pGADT7. The OsFD1 coding sequence was amplified from rice cDNA and inserted into pGADT7 and pGBKT7 vector, respectively. The recombinant plasmids were used for the co-transformation of Y2HGold yeast cells, which were then grown on SD/ − Leu/ − Trp medium for 2–4 days. Single colonies were selected and transferred to SD/ − Leu/ − Trp and SD/ − Trp/ − Leu/ − His/ − Ade media for the subsequent analysis of interactions, which were determined on the basis of colony growth. The primers used for the PCR amplifications are listed in Additional file 4: Table S3.

### Bimolecular Fluorescence Complementation (BiFC) Assay

The *OsFTL4* coding sequence was PCR-amplified and inserted into SCCR vector, and the full-length *Hd3a* sequence was inserted into SCNR vector (Waadt et al. 2008). The OsFTL4/P93L-SCCR mutant vector was obtained by PCR amplification using the OsFTL4/P93L-BD vector as a template. The primers used for the BiFC vector are listed in Additional file 4: Table S3.

### Luciferase Complementation Imaging (LCI) Assay

The full-length sequences of *OsFTL4*, *OsFTL4*/*P93L*, and OsFD1 was inserted into the JW771-nLUC vector, respectively, whereas the *OsGF14* (a to h) and *Nt14-3-3e* coding sequences were inserted into separate JW772-cLUC vector (Gou et al. 2011). The recombinant plasmids were introduced into *A. tumefaciens* strain GV3101 cells, which were then used to infiltrate *N. benthamiana* leaves as previously described (Waadt et al. 2008). Luminescent signals were detected using the Tanon 5200 Chemiluminescent Imaging System. The primers used for the LCI assay are listed in Additional file 4: Table S3.

## Supplementary Information

**Additional file 1. Fig. S1**: Diurnal expression of Ehd1, OsphyB, and OsGI in GLA 4 and the osftl4 mutants under CLD and CSD conditions. **Fig. S2** Protein sequence alignment of 14-3-3 proteins. **Fig. S3** Analysis of OsFTL4 expression in ABA-treated rice seedlings. **Fig. S4** Day length in Yangzhou and Lingshui during the period from sowing to flowering.

**Additional file 2. Table S1**: Comparison of the major agronomic traits of GLA 4 and the osftl4 mutants.

**Additional file 3. Table S2**: Predicted hormone-responsive elements in the OsFTL4 promoter.

**Additional file 4. Table S3**: Primers used in this study.

## Data Availability

The datasets supporting the conclusions of this article are included within the article and its additional files.

## Abbreviations

GLA 4: Guangluai 4
GUS: β-Glucuronidase
qRT-PCR: Quantitative real-time PCR
GFP: Green fluorescent protein
GN: Grain number per panicle
TGW: 1000-grain weight
GYP: Grain yield per plant
CLD: Controlled long-day
CSD: Controlled short-day
NLD: Natural long-day
NSD: Natural short-day
LCI: Luciferase complementation imaging
LUC: Luciferase
PEG: Polyethylene glycol

## References

[CR1] Adachi S, Tsuru Y, Nito N, Murata K, Yamamoto T, Ebitani T, Ookawa T, Hirasawa T (2011). Identification and characterization of genomic regions on chromosomes 4 and 8 that control the rate of photosynthesis in rice leaves. J Exp Bot.

[CR2] Bradley D, Carpenter R, Copsey L, Vincent C, Rothstein S, Coen E (1996). Control of inflorescence architecture in Antirrhinum. Nature.

[CR3] Chardon F, Damerval C (2005). Phylogenomic analysis of the PEBP gene family in cereals. J Mol Evol.

[CR4] Chen S, Tao L, Zeng L, Vega-Sanchez ME, Umemura K, Wang GL (2006). A highly efficient transient protoplast system for analyzing defence gene expression and protein-protein interactions in rice. Mol Plant Pathol.

[CR5] Danilevskaya ON, Meng X, Hou Z, Ananiev EV, Simmons CR (2008). A genomic and expression compendium of the expanded *PEBP* gene family from maize. Plant Physiol.

[CR6] Doi K, Izawa T, Fuse T, Yamanouchi U, Kubo T, Shimatani Z, Yano M, Yoshimura A (2004). *Ehd1* a B-type response regulator in rice, confers short-day promotion of flowering and controls *FT-like* gene expression independently of *Hd1*. Genes Dev.

[CR7] Du H, Huang F, Wu N, Li X, Hu H, Xiong L (2018). Integrative regulation of drought escape through ABA-dependent and -independent pathways in rice. Mol Plant.

[CR8] Fang M, Zhou Z, Zhou X, Yang H, Li M, Li H (2019). Overexpression of *OsFTL10* induces early flowering and improves drought tolerance in *Oryza sativa* L. PeerJ.

[CR9] Galbiati F, Chiozzotto R, Locatelli F, Spada A, Genga A, Fornara F (2016). *Hd3a*, *RFT1* and *Ehd1* integrate photoperiodic and drought stress signals to delay the floral transition in rice. Plant Cell Environ.

[CR10] Gou JY, Felippes FF, Liu CJ, Weigel D, Wang JW (2011). Negative regulation of anthocyanin biosynthesis in *Arabidopsis* by a miR156-targeted SPL transcription factor. Plant Cell.

[CR11] Halliwell J, Borrill P, Gordon A, Kowalczyk R, Pagano ML, Saccomanno B, Bentley AR, Uauy C, Cockram J (2016). Systematic investigation of *FLOWERING LOCUS T*-Like poaceae gene families identifies the short-day expressed flowering Pathway Gene, *TaFT3* in Wheat (*Triticum aestivum* L.). Front Plant Sci.

[CR12] Hiei Y, Ohta S, Komari T, Kumashiro T (1994). Efficient transformation of rice (*Oryza sativa* L.) mediated by *Agrobacterium* and sequence analysis of the boundaries of the T-DNA. Plant J.

[CR13] Hu H, Xiong L (2014). Genetic engineering and breeding of drought-resistant crops. Annu Rev Plant Biol.

[CR14] Hu X, Meng X, Liu Q, Li J, Wang K (2018). Increasing the efficiency of CRISPR-Cas9-VQR precise genome editing in rice. Plant Biotechnol J.

[CR15] Igarashi D, Ishida S, Fukazawa J, Takahashi Y (2001). 14-3-3 proteins regulate intracellular localization of the bZIP transcriptional activator RSG. Plant Cell.

[CR16] Izawa T (2007). Adaptation of flowering-time by natural and artificial selection in *Arabidopsis* and rice. J Exp Bot.

[CR17] Izawa T, Oikawa T, Sugiyama N, Tanisaka T, Yano M, Shimamoto K (2002). Phytochrome mediates the external light signal to repress *FT* orthologs in photoperiodic flowering of rice. Genes Dev.

[CR18] Kaneko-Suzuki M, Kurihara-Ishikawa R, Okushita-Terakawa C, Kojima C, Nagano-Fujiwara M, Ohki I, Tsuji H, Shimamoto K, Taoka K-I (2018). TFL1-Like proteins in rice antagonize rice FT-Like protein in Inflorescence development by competition for complex formation with 14-3-3 and FD. Plant Cell Physiol.

[CR19] Karlgren A, Gyllenstrand N, Källman T, Sundström JF, Moore D, Lascoux M, Lagercrantz U (2011). Evolution of the PEBP gene family in plants: functional diversification in seed plant evolution. Plant Physiol.

[CR20] Kobayashi K, Yasuno N, Sato Y, Yoda M, Yamazaki R, Kimizu M, Yoshida H, Nagamura Y, Kyozuka J (2012). Inflorescence meristem identity in rice is specified by overlapping functions of three *AP1/FUL*-like MADS box genes and *PAP2*, a *SEPALLATA* MADS box gene. Plant Cell.

[CR21] Kojima S, Takahashi Y, Kobayashi Y, Monna L, Sasaki T, Araki T, Yano M (2002). *Hd3a*, a rice ortholog of the *Arabidopsis FT* gene, promotes transition to flowering downstream of *Hd1* under short-day conditions. Plant Cell Physiol.

[CR22] Komiya R, Ikegami A, Tamaki S, Yokoi S, Shimamoto K (2008). *Hd3a* and *RFT1* are essential for flowering in rice. Development.

[CR23] Komiya R, Yokoi S, Shimamoto K (2009). A gene network for long-day flowering activates *RFT1* encoding a mobile flowering signal in rice. Development.

[CR24] Kumar S, Stecher G, Li M, Knyaz C, Tamura K (2018). MEGA X: Molecular evolutionary genetics analysis across computing platforms. Mol Biol Evol.

[CR25] Kusumi K, Hirotsuka S, Kumamaru T, Iba K (2012). Increased leaf photosynthesis caused by elevated stomatal conductance in a rice mutant deficient in SLAC1, a guard cell anion channel protein. J Exp Bot.

[CR26] Lifschitz E, Ayre BG, Eshed Y (2014). Florigen and anti-florigen: a systemic mechanism for coordinating growth and termination in flowering plants. Front Plant Sci.

[CR27] Nakagawa M, Shimamoto K, Kyozuka J (2002). Overexpression of *RCN1* and *RCN2*, rice *TERMINAL FLOWER 1*/*CENTRORADIALIS* homologs, confers delay of phase transition and altered panicle morphology in rice. Plant J.

[CR28] Nakamura S, Abe F, Kawahigashi H, Nakazono K, Tagiri A, Matsumoto T, Utsugi S, Ogawa T, Handa H, Ishida H, Mori M, Kawaura K, Ogihara Y, Miura H (2011). A wheat homolog of MOTHER OF FT AND TFL1 acts in the regulation of germination. Plant Cell.

[CR29] Nemoto Y, Nonoue Y, Yano M, Izawa T (2016). *Hd1*, a* CONSTAN*S ortholog in rice, functions as an* Ehd*1 repressor through interaction with monocot-specific CCT-domain protein Ghd7. Plant J.

[CR30] Pasriga R, Cho LH, Yoon J, An G (2018). Identification of the regulatory region responsible for vascular tissue-specific expression in the Rice *Hd3a* promoter. Mol Cells.

[CR31] Preston JC, Kellogg EA (2006). Reconstructing the evolutionary history of paralogous *APETALA1*/*FRUITFULL*-Like genes in grasses (Poaceae). Genetics.

[CR32] Rabbani MA, Maruyama K, Abe H, Khan MA, Katsura K, Ito Y, Yoshiwara K, Seki M, Shinozaki K, Yamaguchi-Shinozaki K (2003). Monitoring expression profiles of rice genes under cold, drought, and high-salinity stresses and abscisic acid application using cDNA microarray and RNA gel-blot analyses. Plant Physiol.

[CR33] Riboni M, Galbiati M, Tonelli C, Conti L (2013). *GIGANTEA* enables drought escape response via abscisic acid-dependent activation of the florigens and *SUPPRESSOR OF OVEREXPRESSION OF CONSTANS*. Plant Physiol.

[CR34] Shim JS, Jang G (2020). Environmental signal-dependent regulation of flowering time in rice. Int J Mol Sci.

[CR35] Sohn EJ, Rojas-Pierce M, Pan S, Carter C, Serrano-Mislata A, Madueño F, Rojo E, Surpin M, Raikhel NV (2007). The shoot meristem identity gene *TFL1* is involved in flower development and trafficking to the protein storage vacuole. Proc Natl Acad Sci USA.

[CR36] Song YH, Shim JS, Kinmonth-Schultz HA, Imaizumi T (2015). Photoperiodic flowering: time measurement mechanisms in leaves. Annu Rev Plant Biol.

[CR37] Sun C, Chen D, Fang J, Wang P, Deng X, Chu C (2014). Understanding the genetic and epigenetic architecture in complex network of rice flowering pathways. Protein Cell.

[CR38] Tamaki S, Matsuo S, Wong HL, Yokoi S, Shimamoto K (2007). Hd3a protein is a mobile flowering signal in rice. Science.

[CR39] Tamaki S, Tsuji H, Matsumoto A, Fujita A, Shimatani Z, Terada R, Sakamoto T, Kurata T, Shimamoto K (2015). FT-like proteins induce transposon silencing in the shoot apex during floral induction in rice. Proc Natl Acad Sci USA.

[CR40] Taoka K, Ohki I, Tsuji H, Furuita K, Hayashi K, Yanase T, Yamaguchi M, Nakashima C, Purwestri YA, Tamaki S, Ogaki Y, Shimada C, Nakagawa A, Kojima C, Shimamoto K (2011). 14–3-3 proteins act as intracellular receptors for rice Hd3a florigen. Nature.

[CR41] Taoka K, Ohki I, Tsuji H, Kojima C, Shimamoto K (2013). Structure and function of florigen and the receptor complex. Trends Plant Sci.

[CR42] Vaistij FE, Barros-Galvão T, Cole AF, Gilday AD, He Z, Li Y, Harvey D, Larson TR, Graham IA (2018). *MOTHER-OF-FT-AND-TFL1* represses seed germination under far-red light by modulating phytohormone responses in *Arabidopsis thaliana*. Proc Natl Acad Sci.

[CR43] Valim HF, McGale E, Yon F, Halitschke R, Fragoso V, Schuman MC, Baldwin IT (2019). The clock gene *TOC1* in shoots, not roots, determines fitness of *Nicotiana attenuata* under drought. Plant Physiol.

[CR44] Waadt R, Schmidt LK, Lohse M, Hashimoto K, Bock R, Kudla J (2008). Multicolor bimolecular fluorescence complementation reveals simultaneous formation of alternative CBL/CIPK complexes in planta. Plant J.

[CR45] Wang Y, Lu Y, Guo Z, Ding Y, Ding C (2020). *RICE CENTRORADIALIS 1*, a *TFL1*-like gene, responses to drought stress and regulates rice flowering transition. Rice (NY).

[CR46] Wei FJ, Tsai YC, Wu HP, Huang LT, Chen YC, Chen YF, Wu CC, Tseng YT, Hsing YC (2016). Both *Hd1* and *Ehd1* are important for artificial selection of flowering time in cultivated rice. Plant Sci.

[CR47] Xi W, Liu C, Hou X, Yu H (2010). *MOTHER OF FT AND TFL1* regulates seed germination through a negative feedback loop modulating ABA signaling in *Arabidopsis*. Plant Cell.

[CR48] Xiao B, Huang Y, Tang N, Xiong L (2007). Over-expression of a *LEA* gene in rice improves drought resistance under the field conditions. Theor Appl Genet.

[CR49] Xu Y-F, Ookawa T, Ishihara K (1997). Analysis of the photosynthetic characteristics of the high-yielding rice cultivar Takanari. Jpn J Crop Sci.

[CR50] Yang Y, Peng Q, Chen GX, Li XH, Wu CY (2013). OsELF3 is involved in circadian clock regulation for promoting flowering under long-day conditions in rice. Mol Plant.

[CR51] Zhan Z, Zhang C, Zhang H, Li X, Wen C, Liang Y (2017). Molecular cloning, expression analysis, and subcellular localization of *FLOWERING LOCUS T* (*FT*) in carrot (*Daucus carota* L.). Mol Breeding.

[CR52] Zhang Y, Su J, Duan S, Ao Y, Dai J, Liu J, Wang P, Li Y, Liu B, Feng D, Wang J, Wang H (2011). A highly efficient rice green tissue protoplast system for transient gene expression and studying light/chloroplast-related processes. Plant Methods.

[CR53] Zhang C, Liu J, Zhao T, Gomez A, Li C, Yu C, Li H, Lin J, Yang Y, Liu B, Lin C (2016). A drought-inducible transcription factor delays reproductive timing in rice. Plant Physiol.

[CR54] Zhang S, Jin Y, Hao H, Liang S, Ma X, Luan W (2020). Characterization and identification of *OsFTL8* gene in rice. Plant Biotechnol Rep.

[CR55] Zhu G, Ye N, Zhang J (2009). Glucose-induced delay of seed germination in rice is mediated by the suppression of ABA catabolism rather than an enhancement of ABA biosynthesis. Plant Cell Physiol.

[CR56] Zong W, Tang N, Yang J, Peng L, Ma S, Xu Y, Li G, Xiong L (2016). Feedback regulation of ABA signaling and biosynthesis by a bZIP transcription factor targets drought-resistance-related genes. Plant Physiol.

