# Supplementary material for: OsFTL4, an FT-like Gene, Regulates Flowering Time and Drought Tolerance in Rice (Oryza sativa L.)
Source: Rice (N Y). 2022 Sep 6;15:47. doi: 10.1186/s12284-022-00593-1 (PMC9448835; doi:10.1186/s12284-022-00593-1)
Supplement: Supplementary file 1 — Additional file 1. Fig. S1: Diurnal expression of Ehd1, OsphyB, and OsGI in GLA 4 and the osftl4 mutants under CLD and CSD conditions. Fig. S2 Protein sequence alignment of 14-3-3 proteins. Fig. S3 Analysis of OsFTL4 expression in ABA-treated rice seedlings. Fig. S4 Day length in Yangzhou and Lingshui during the period from sowing to flowering. [file 12284_2022_593_MOESM1_ESM.docx]

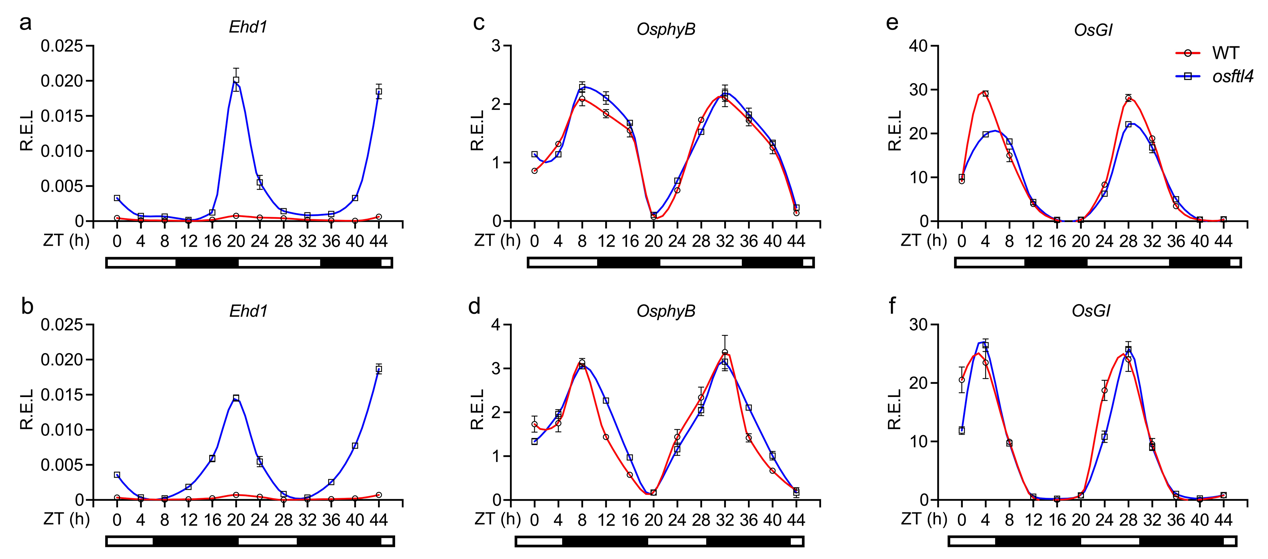


**Fig. S1** Diurnal expression of *Ehd1*, *OsphyB*, and *OsGI* in GLA 4 wild-type and the *osftl4* mutants under CLD and CSD conditions. CLDs (a, c, and e); CSDs (b, d, and f). R. E. L: Relative expression level. The open and filled bars at the bottom represent the light and dark periods, respectively. The rice *OsActin* gene was used as the internal control. Data are shown as mean ± SD of three independent experiments and three biological replicates. *osftl4-1* was used for circadian pattern analysis.


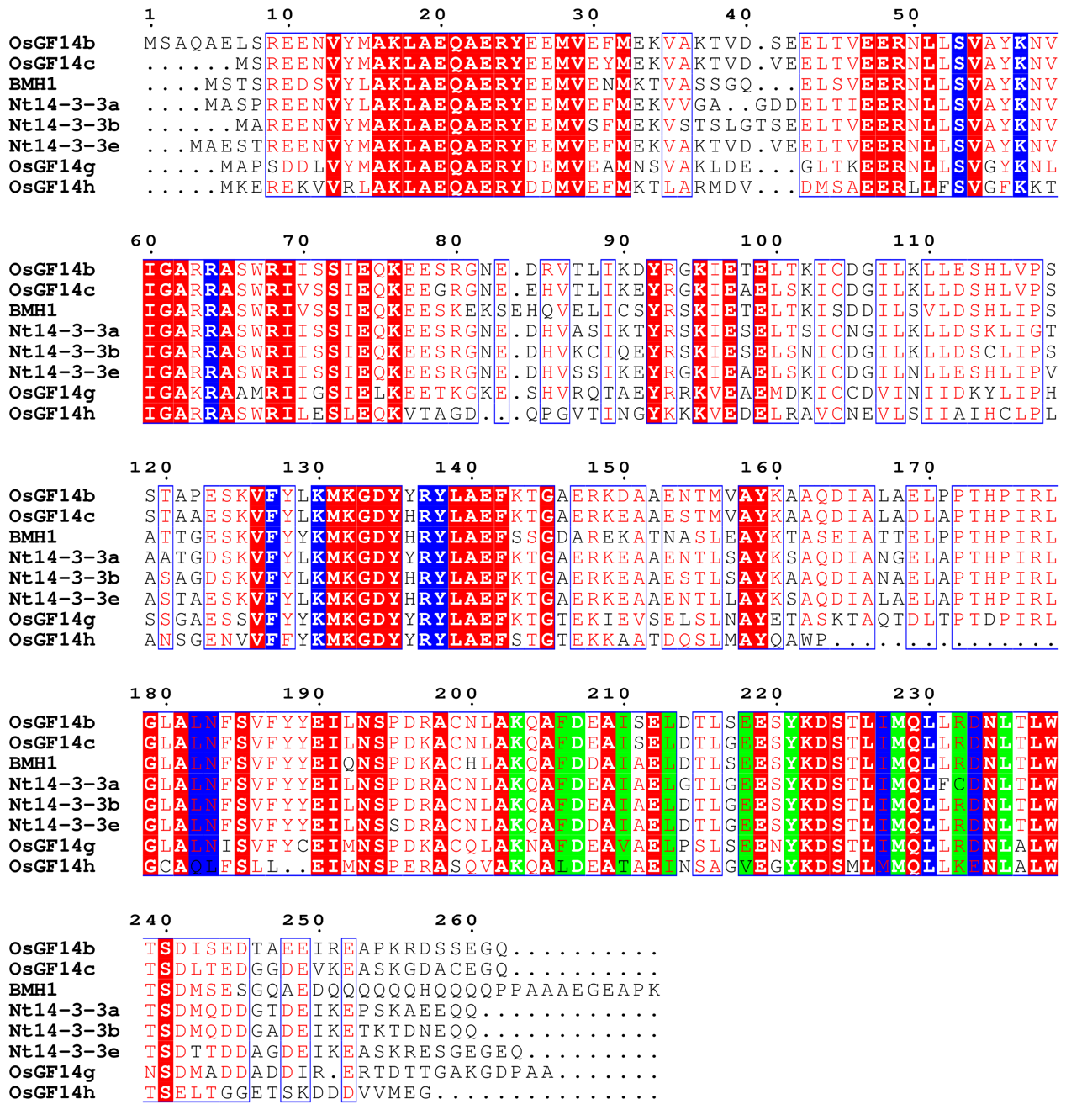


**Fig. S2** Protein sequence alignment of 14-3-3 proteins. Residues in a green background are located at the Hd3a binding site, and are conserved between yeast, tobacco, and rice. Those on a blue background interact with OsFD1.


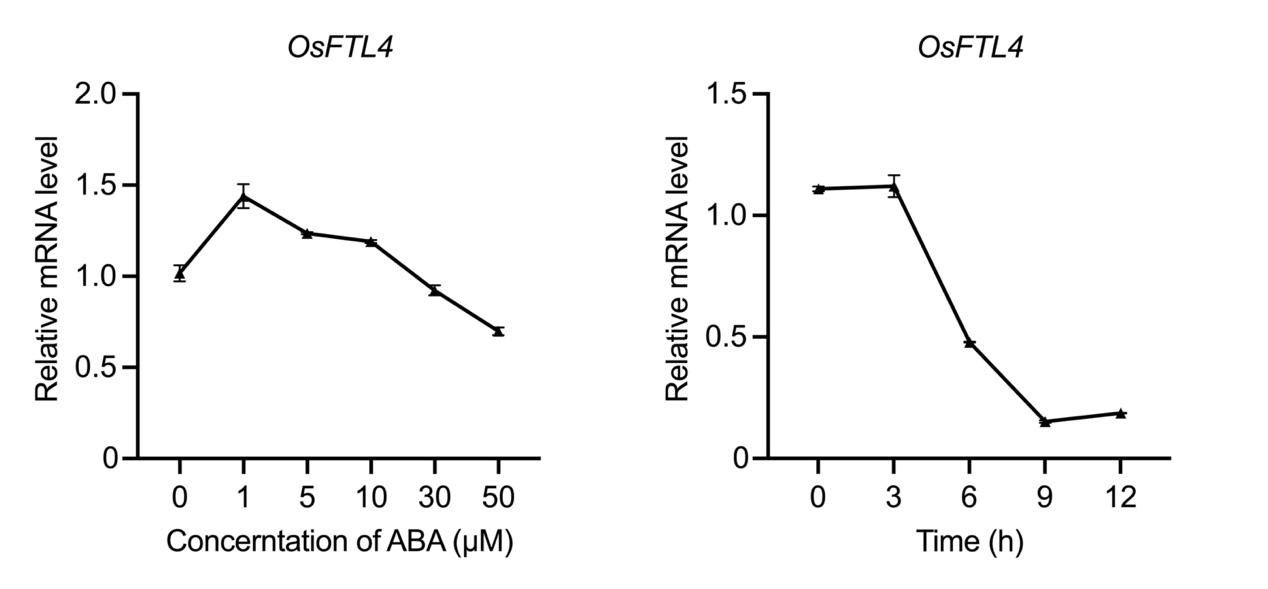


**Fig. S3** Analysis of *OsFTL4* expression in ABA-treated rice seedlings. Relative mRNA level of *OsFTL4* was normalized to *OsActin*. RNA was extracted for expression analysis at 3-h after ABA treatment (left) and at 3-h interval after treatment of 10uM ABA (right). Values are shown as mean ± SD of three independent experiments and three biological replicates.


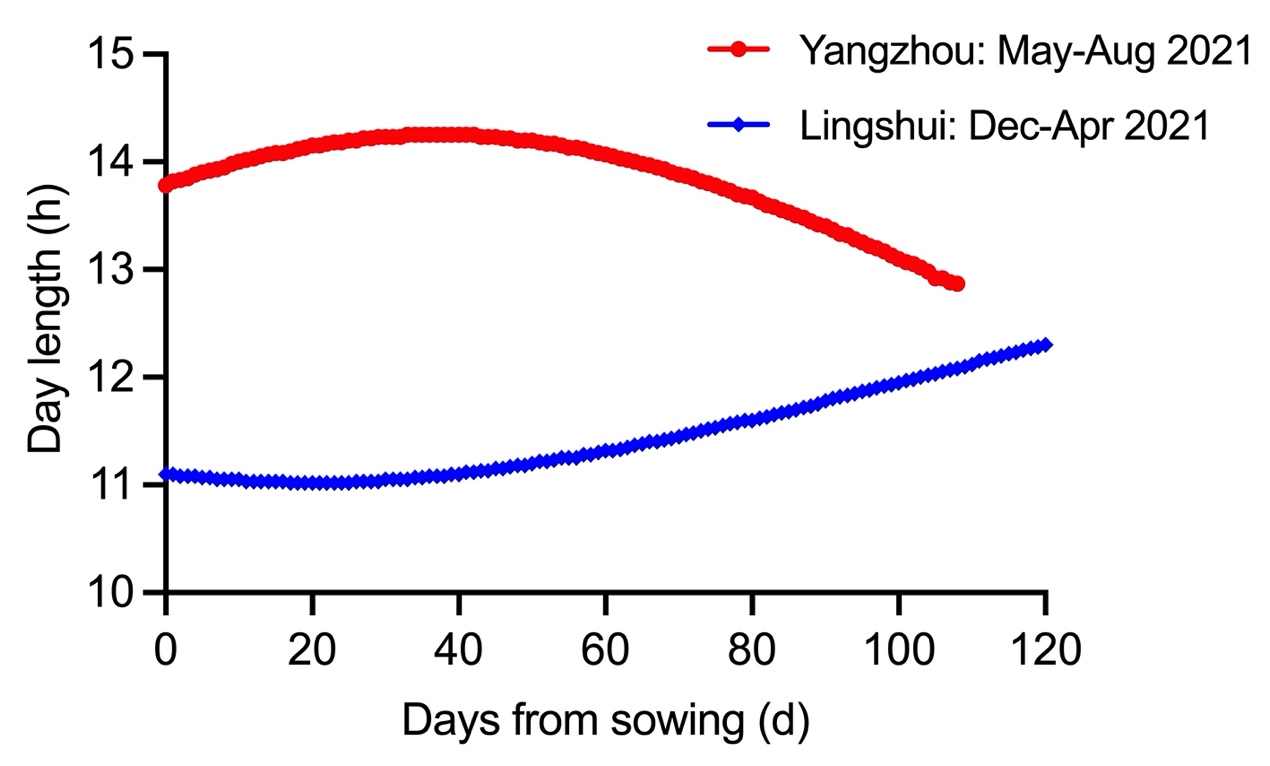


**Fig. S4** Day length in Yangzhou and Lingshui during the period from sowing to flowering.
